# Change in the immune function of porcine iliac artery endothelial cells infected with porcine circovirus type 2 and its inhibition on monocyte derived dendritic cells maturation

**DOI:** 10.1371/journal.pone.0186775

**Published:** 2017-10-26

**Authors:** Ning Yang, Jinzeng Qiao, Shiyu Liu, Zhanming Zou, Linlin Zhu, Xinyu Liu, Shuanghai Zhou, Huanrong Li

**Affiliations:** College of Animal Science and Technology, Beijing Key Laboratory of Traditional Chinese Veterinary Medicine, Beijing University of Agriculture, Beijing, P. R., China; Centre National de la Recherche Scientifique, FRANCE

## Abstract

Porcine circovirus-associated disease is caused by porcine circovirus type 2 (PCV2) infection, which targets iliac artery endothelial cells (PIECs); it leads to severe immunopathologies and is associated with major economic losses in the porcine industry. Here, we report that in vitro PCV2 infection of PIECs causes cell injury, which affects DC function as well as adaptive immunity. Specifically, PCV2 infection downregulated PIEC antigen-presenting molecule expression, upregulated cytokines involved in the immune and inflammatory response causing cell damage and repair, and altered the migratory capacity of PIECs. In addition, PCV2-infected PIECs inhibited DC maturation, enhanced the endocytic ability of DCs, and weakened the stimulatory effect of DCs on T lymphocytes. Together, these findings indicate that profound functional impairment of DCs in the presence of PCV2-infected PIECs may be a potential pathogenic mechanism associated with PCV2-induced porcine disease.

## Introduction

Porcine circovirus type 2 (PCV2) is the causative agent of PCV2-associated disease (PCVAD), which leads to immense economic losses in the porcine industry worldwide [1–3]. There is considerable evidence for the various presentations of PCV2 infection, such as porcine dermatitis and nephropathy syndrome, reproductive failure, proliferative and necrotizing pneumonia, respiratory disease and enteritis [4]. In particular, immunosuppression and immune injury are the hallmarks of PCV2 infection and PCVAD [2, 5, 6].

Research in the recent decade has highlighted the involvement of vascular lesions/alterations in the pathogenesis of certain PCVD presentations. For example, PCVD-infected swine exhibit blood hypercoagulation, petechiae and vasculitis associated with lymph node atrophy, organ failure with ischemic, necrotic lesions, and brain hemorrhage [7, 8]. PCV2 not only causes severe degeneration of endothelial cells (ECs) [9], but also stimulates procoagulant activity in ECs and leads to vascular injury [8, 10], which indicates that the hemostatic system and ECs play an important role in the immune pathogenesis of PCV2. Naturally infected PCV2 swine exhibit endarteritis, and the PCV2 antigen has been detected in vascular endothelial cells (VECs) in infected swine [11]. PCV2 has a direct cytopathic effect on tunica media myocytes of small- and medium-sized arteries as well as the endothelium [12]. Moreover, PCV2 infection can influence VEC function by upregulating the expression of endothelial adhesion and junction molecules [10], and Th1 and Th2 cytokines [13].

VECs can affect the innate and acquired immune response, inflammatory response, coagulation and angiogenesis by regulating leukocyte transport, production of inflammatory cytokines and chemokines, expression of antigen-presenting molecules related to MHCII, and so on [14–19]. Cytokines secreted by human umbilical vein ECs can affect the immune response by inhibiting the differentiation of monocytes to dendritic cells (DCs) [20]. Endothelial IL-8, IL-6 and VEGF can inhibit the maturation of DCs [21–23]. And DCs are the most potent antigen-presenting cells (APCs), which can activate naive T lymphocytes and initiate adaptive immune responses [24]. The maturation and function of DCs depend on its adhesion with VECs and migration through the vascular wall [25]. Accordingly, any change in the micro-environment during migration may affect the maturation of DCs and their immune function. So, it would be interesting to study changes in the immune function of PCV2-infected VECs and its effects on DCs.

The aim of this study was to characterize changes in VEC immune function after PCV2 infection by analyzing the immune-related gene and cytokine profiles and migration capacity in porcine iliac artery endothelial cells (PIECs) infected with PCV2. Moreover, the impact of the infection on the differentiation, maturation and antigen presentation function of monocyte-derived DCs was also examined.

## Materials and methods

### Virus and cells

PCV2-SD/2008 (GenBank accession number: GQ174519) was isolated and identified by the Laboratory of Animal Infectious Diseases at Hebei Agricultural University. The isolate and the methods used for identification of PCV2 were described previously [26]. The lysate obtained after the third passage of PCV2 through the PCV1/PCV2-free PK-15 cells was used as virus stock, and the titer was 10^5.5^ TCID_50_/mL (TCID_50_ = 50% tissue culture infectious dose), according to the Reed-Muench assay.

PIECs obtained from the Cell Resource Center of Shanghai Institutes for Biological Sciences (Shanghai, China, Catalog number: GN105) were maintained in RPMI 1640 (GIBICO, Grand land, NY, USA) supplemented with 10% heat-inactivated fetal bovine serum (FBS) (Sigma, Missouri, USA) and 200 U of penicillin-streptomycin/ml at 37°C in a humidified 5% CO_2_ incubator (Thermo, New York, USA).

### Animals

Six healthy, 21-day-old, large white weaning piglets were obtained from the Beijing Centre of SPF Swine Breeding and Management, which is located in the East of Che Er Ying Village, Nie Ge Zhuang Township, Haidian District, Beijing. This farm has been checked and certified by the Beijing Science and Technology Committee, and its unified social credit code is 12110000400685619H. Mycoplasma pneumonia of swine, swine dysentery, infectious swine atrophic rhinitis, porcine pseudorabies, swine transmissible gastroenteritis, lice and mites were not detected during the establishment of the SPF swine population. These purchased animals were also confirmed to be free of PCV2, porcine circovirus type 1 (PCV1), porcine reproductive and respiratory syndrome virus (PRRSV), porcine parvovirus (PPV), and classical swine fever virus (CSFV) by PCR/RT-PCR. The feeding, housing and husbandry practices were in accordance with the animal welfare requirements of the Beijing Administration Office of Laboratory Animal Care and Ethics Committee. Animals were raised in isolated rooms with individual ventilation and received food and water ad libitum. At the end of the experiment, pigs were euthanized with an overdose of pentobarbital-sodium (70–80 mg/kg; Sinopharm Chemical Reagent Beijing Co., Beijing, China) injected intravenously.

Blood samples (10 ml) treated with ethylene diamine tetraacetic acid (EDTA) from the front cavity vein of each piglet were collected for isolating peripheral blood mononuclear cells (PBMCs), under the ethical approval of the Beijing University of Agriculture Animal Science and Technology College (Approval Number SYXK (BUA) 2015–0006). The protocol was approved by the Beijing Administration Office of Laboratory Animal Care and Ethics Committee, China.

### Virus infection and sample collection

PIECs were seeded (4×10^5^ cells/well) into a 6-well cell culture plate. Once the cells reached approximately 50–70% confluence, the culture medium was removed from the wells and cells were washed three times with PBS. The PIECs were infected with PCV2 SD/2008 at a multiplicity of infection (MOI) of 0.5 for 1 h. The control cells were treated with the same volume of RPMI 1640. All the experiments were performed in triplicate. The infection medium was washed away and the cells were incubated in humidified 5% CO_2_ atmosphere at 37°C with 2% FBS RPMI 1640. The cells and supernatants were collected at different time after infection for further experiments.

### RNA extraction and first-strand cDNA synthesis

RNA was extracted using the TRIzol LS Reagent (Invitrogen, California, USA), according to the manufacturer’s instructions. Single-stranded cDNA was synthesized from 2 μg of total RNA using the HiFi-MMLV cDNA kit (Cowin Biotech, Beijing, China).

### Real-time fluorescence quantitative PCR

The real-time fluorescence quantitative PCR (FQ-PCR) procedure was in accordance with previous reports [27]. It was performed with an Mx3005P real-time qPCR system (Agilent, California, USA) and Ultra SYBR Mixture (Cwbiotech, Beijing, China), using a 20-μl reaction solution containing 10 μl 2× Ultra SYBR mixture (Cwbiotech, Bengjing, China), 0.5 μl of each primer (Table 1), 2 μl DNA template and 7 μl DNase/RNase-free water. The thermal cycler profile was 95°C for 10 min, followed by 40 cycles of 95°C for 30 s, 50°C for 30 s, and 72°C for 30 s. All samples were measured in triplicate. Differences in gene expression were calculated using the 2-cycle threshold method. β-actin was used as a reference gene. For analysis of mRNA expression of each validated gene, raw data were normalized against the values obtained for β-actin mRNA, and the fold changes in the expression of each gene in the infected group vs. the control group were determined. The standard curve for PCV2 was obtained using 10-fold dilutions of plasmid DNA for viral loads.

**Table 1:** Swine-specific primer sequences used for quantitative SYBR1 ROX-1 real-time PCR.

Target gene	Accession number	Primer sequence	Product (bp)	Annealing temperature (°C)
**IL-8**	NM_213867	For: 5′-TCCTGCTTTCTGCAGCTCTC-3′	100	52
		Rev: 5′-GGGTGGAAAGGTGTGGAATG-3′		
**CCL2**	NM_214214	For: 5′-GAAGAGTCACCAGCAGCAAG-3′	112	53
		Rev: 5′-TGGCTTATGGAGTCCTGGAC-3′		
**CCL4**	NM_213779	For: 5′-ATGAAGCTCTGCGTGACTGT-3′	117	55
		Rev: 5′-GGTGTATGTGAAGCAGCAGG-3′		
**AMCF-II**	NM_213876	For: 5′- GTGTTTAACCACCACACCCG-3′	113	54
		Rev: 5′-TTCCATTCTTCAGGGTGGCT-3′		
**SLA-DQA1**	NM_001130224	For: 5′- GCCACTTCTGAAACACTGGG-3′	138	55
		Rev: 5′- CGCAGGCCTTGAATGATGAA-3′		
**SLA-DMA**	NM_001113705	For: 5′-TTTCTCCTGCGTCGTGACTC-3′	138	56
		Rev: 5′-ATGATTCCCAGCACACCCAG-3′		
**SLA-DMB**	NM_001113707	For: 5′-ACATACCAGACCGTCTCCCA-3′	119	54
		Rev: 5′-GACAGCCCAGAAGTCCAGTC-3′		
**β-actin**	U07786	For: 5′-CTGGCATTGTCATGGACTCT-3′	547	57
		Rev: 5′-GCGATGATCTTGATCTTCAT-3′		
**PCV2**	JN176181	For: 5′-TGCCAGTTCGTCACCCTTT-3′	188	50
		Rev: 5′-CAGTATATACGACCAGGACTACAATATC-3′		

Note: β-actin was used as the internal control.

bp: base pairs; for: forward primer; rev: reverse primer.

### Detection of viral loads of PCV2-infected PIECs by real-time FQPCR

PIECs were infected with PCV2 at an MOI of 0.5, and then the mixture of cells and supernatant or cells was collected at 4, 12, 24, 48 and 72 h post infection (hpi). Viral DNA was extracted at different time points using the TIANamp genomic DNA kit (TIANGEN, Beijing, DP) according to the manufacturer’s instruction. The viral loads of PCV2 in the mixture and PIECs were determined using real-time FQPCR.

### Detection of apoptosis of PCV2-infected PIECs by flow cytometry

Cell apoptosis assay was carried out in accordance with the previously reported method [28]. as follows: Apoptosis of PCV2-infected PIECs was detected with the Annexin V-FITC apoptosis detection kit (Beyotime Institute of Biotechnology, Beijing, China) according to the manufacturer’s instructions. PCV2-infected PIECs and control PIECs (untreated) were collected at 4, 12, 24, 48, and 72 hpi, washed twice with PBS, and stained with Annexin V-FITC/PI at room temperature in the dark for 15 min. Finally, the cells were assessed with ACEA NovoCyte^TM^ flow cytometry, and the percentage of apoptotic cells was determined using the NovoExpresss software.

### Detection of IL-8 in PCV2-infected PIECs

Changes in the expression levels of the cytokine IL-8 from PCV2-infected PIECs and cell culture supernatants collected at 4, 12, 24, 48 and 72 hpi were analyzed by real-time FQPCR and enzyme-linked immunosorbent assay (ELISA) (R&D, Minnesota, USA), respectively. ELISA was performed in accordance with the manufacturer’s instructions. All samples were measured in triplicate. The plates were read using an ELISA reader (Bio-Tek, Vermont, USA) at 450 nm. A standard curve was generated from two-fold serial dilutions of porcine IL-8. Based on the standard curve, the concentration of each unknown sample was calculated from the OD value and the data were expressed as the mean concentration in ng/mL.

### Analysis and validation of the gene chip used to measure changes in the immune function of PCV2-infected PIECs

PCV2-infected PIECs detected by microarray were collected at different time points according to the change in viral load and related functional molecules. Total RNA was isolated from the PCV2-infected PIECs and control PIECs (three samples per group) for amplifying and labeling with Cy-3 using the low Input Quick Amp Labeling Kit, One-Color (Agilent Technologies, California, USA), according to the manufacturer’s instructions. Labeled cRNA was purified using the RNeasy mini kit (QIAGEN, GmBH, Germany). Each array slide was hybridized with 1.65ug of Cy3-labeled cRNA using the Gene Expression Hybridization kit (Agilent Technologies, California, USA) in a hybridization oven. Slides were scanned using the Agilent Microarray Scanner (Agilent Technologies, California, USA). Data were extracted with Feature Extraction software 10.7 (Agilent Technologies, California, USA). Raw data were normalized using the Quantile algorithm for statistical analysis in the Gene Spring Software. Gene Sping GX was used to screen for multiple differentially expressed genes, such as those involved in immune responses, inflammation, injury repair, antigen processing and presentation, chemotaxis, and migration (Table 2). The functions of the differentially expressed genes were analyzed using the bioinformatics resources Database for Annotation, Visualization and Integrated Discovery (DAVID 6.6; GO).

**Table 2:** Gene ontology functional classification of genes that are differentially regulated in PIECs following PCV2 infection.

Item	n	Gene
Immune response	24	↑: OAS1. OAS2. DDX58. AMCF-II. CCL2. CCL20. CCL4.CSF2.C1S.IL1A.IL12A.IL18.IL8
		↓: CFB. CD70. SLA-DRB1. SLA-DMB. SLA-DQA1. SLA-DMA. SLA-DQB1. C5. GBP1. TLR1. TNFSF10
Inflammatory response	7	↑: AMCF-II. ITIH4. IL8. IL1A
		↓: CFB. FTUIN. C5
Response to wounding	11	↑: C1S. CCL4. ITIH4. AMCF-II. IL8. CCL2
		↓: FETUIN. CFB. C5. PROC. THBD
Antigen processing and presentation of peptides	5	↓: SLA-DRB1. SLA-DMB. SLA-DQA1. SLA-DMA. SLA-DQB1
Chemotaxis/Taxis/Locomotion behaviors	4	↑: AMCF-II. CCL2. CCL4. IL8

Note: *P* < 0.05 compared to uninfected PIECs for all genes

PIECs: Porcine iliac artery endothelial cells

To validate the microarray results, the relative quantities of selected genes, such as those involved in antigen processing and presentation of peptide via MHC class II (SLA-DQA1, SLA-DMA, and SLA-DMB), immune response (IL-8, CCL2, CCL4, AMCF-II, SLA-DQA1, SLA-DMA, and SLA-DMB), inflammatory response (AMCF-II and IL-8), response to wounding (AMCF-II, IL-8, CCL2, and CCL4), and behavior (IL-8, CCL2, CCL4, and AMCF-II), were analyzed by real-time FQPCR. The primer sets used to measure the expression levels of the different cytokines are reported in Table 1.

### PIEC migration assay

PIEC migration was determined as previously described [29] with minor modifications. PIECs were seeded (4×10^5^cells/well) into 6-well plates and cultured for 24 h under static conditions. The confluent EC monolayer was scraped in a straight line with a 200 μL pipette tip to create a wound parallel to the direction of the flow. Wounded monolayers were washed once with 1 mL of growth medium to remove cell debris. The in vitro scratch wound healing assay was performed under four different conditions including a control group, PCV2-infected group, IL-8-antibody group, and PCV2-infected-IL-8-antibody group (n = 3 wells per group). PCV2-infected groups were infected as described above. IL-8 antibody was added into the cell supernatants at a final concentration of 3 µg/mL (Abcam, Cambridge, UK) to the relevant groups. The cells were grown in a humidified 5% CO_2_ atmosphere at 37°C for 24 h. Images of the cell monolayer around the wounds were taken with an Olympus inverted phase microscope at 24 hpi (Olympus, Tokyo, Japan).

Data were presented as wound closure (%): Wound closure [(A0 -At)/A_0_]×100% where A_0_ is the area of the original wound and A_t_ is the area of wound measured 24 hpi. All experiments were repeated at least three times.

### Purification of CD14^+^ monocytes from PBMCs

PBMCs were isolated from buffy coats collected from SPF pigs by density centrifugation over Ficoll-Paque (1.077 g/L) at 400 × *g* for 20 min. CD14^+^ monocytes (FITC-CD14, Bio-Rad, California, USA) were selected by magnetic cell sorting using MACS (Miltenyi, Bergisch-Gladbach, Germany). The isolated monocytes were stained with trypan blue and the concentration was adjusted to 1 × 10^6^ cells/mL. According to previous studies [30] and our pre-experimental results, 10% FBS RP1640 medium containing 5 ng/mL porcine granulocyte-macrophage colony-stimulating factor (GM-CSF) (R&D, Minnesota, USA) and 20 ng/mL recombinant porcine IL-4 (ProSpec, Rehovot, Israel) was used to induce monocyte-derived DCs in a humidified 5% CO_2_ atmosphere at 37°C for 5 days.

### Co-culture of PIECs and DCs

Transwells (1.0-μm pore size; Millipore, Massachusetts, USA) were suspended in 24-well cell culture plates (Corning, New York, USA). Monocytes were seeded into the upper reservoir of the transwell chamber in 10% FBS RPMI1640 containing recombinant porcine GM-CSF and recombinant porcine IL-4. DCs were allowed to develop in the upper chamber of the transwell, and half of the medium was changed every other day for 5 days. The resulting immature dendritic cells (imDCs) were then co-cultured with PIECs that were PCV2-infected or non-infected PIECs for 12, 24, 36, and 48 h in a humidified 5% CO_2_ atmosphere at 37°C. Each time point was performed in triplicate and three independent experiments were performed (n = 3). The ratio between imDC and PIEC cells was 1:10. Four groups were used in each experiment: (1) co-culture control group (PIEC-DC), (2) co-culture PCV2-infected PIEC group (PCV2-PIEC-DC), (3) co-culture PCV2-infected DC group (PCV2-DC), and (4) DCs alone. In the co-culture groups, the PIECs were placed in the lower chamber of the transwell and the imDCs were in the upper chamber. The cells in the upper chambers were characterized by flow cytometry and functionally assayed.

### Detection of monocytes and markers of DC by flow cytometry

Induced DCs from the upper chamber of each transwell were collected and suspended in 250 µL of PBS. DCs stained with the FITC-anti-CD1a antibody and R-PE-anti-SWC3a antibody (Beckman Coulter, Florida, USA) were re-suspended in PBS and analyzed using a flow cytometer (EPICS ELITE; Beckman Coulter, Florida, USA). Cells were stained with the FITC-labeled anti-SLA-DR antibody (AbD Serotec, Kidlington, UK) and the PE-labeled anti-CD80/86 antibody (Ancell, California, USA) for 30 min at room temperature in the dark. The cells were then rinsed with PBS, resuspended in PBS, and then detected by flow cytometry.

CD1a and SWC3a double-positive cells indicated the presence of DCs. Effects of PCV2-infected PIEC on CD80/86 and MHC-II expression on DCs were analyzed. In addition, the data collected from the flow cytometry experiments were used to determine the timing of the subsequent DC endocytosis and antigen presentation assays.

### DC endocytosis assay

DCs were collected and 150 μL of each sample cell suspension was incubated with 150 μL of FITC-Dextran (Sigma, Missouri, USA, 40KD molecular weight, 1 mg/ml) in a humidified 5% CO_2_ atmosphere for 1h at 37 °C, then washed with PBS. Endocytic activity of DCs stimulated by dextran was quantified by measuring the percentage of FITC^+^ DCs by flow cytometry.

### T-cell proliferation assay

For proliferation assays, T cells were enriched from the PBMCs of allogeneic animals using a nylon wool column (Nylon Fiber Column T (L-Type), Wako, Japan). 2×10^6^ T cells/ml were mixed with 2×10^5^ DCs/ml in 96-well cell culture plates at a ratio of 10:1. T cells without DCs were used as the control. The cultures were incubated in growth medium at 37°C, 5% CO_2_ for 3 days. Cell proliferation assay using the CellTiter 96^®^ AQ_ueous_ One Solution (Promega, Wisconsin, USA) was performed according to the manufacturer’s instructions. In brief, 20 μL of the CellTiter 96^®^ AQ_ueous_ One Solution was added to each well and incubated for 4 h. The optical density (OD 490 nm) was recorded using a microplate reader (Bio-Rad, California, USA). The stimulation index was calculated from the raw data using the following formula:

$$\left(SI\right)=(OD_{treated}-OD_{blank})/(OD_{untreated}-OD_{blank}).$$

### Statistical analysis

The data depicted in each figure is from one representative experiment out of at least three independently performed experiments. A one-way ANOVA was used to determine whether the differences between groups were statistically significant. *P* values < 0.05 were considered to indicate significant differences, and those <0.01 were considered to indicate very significant differences.

## Results

### Replication and nucleic acid dynamics of PCV2 in PIECs

**Fig 1:**
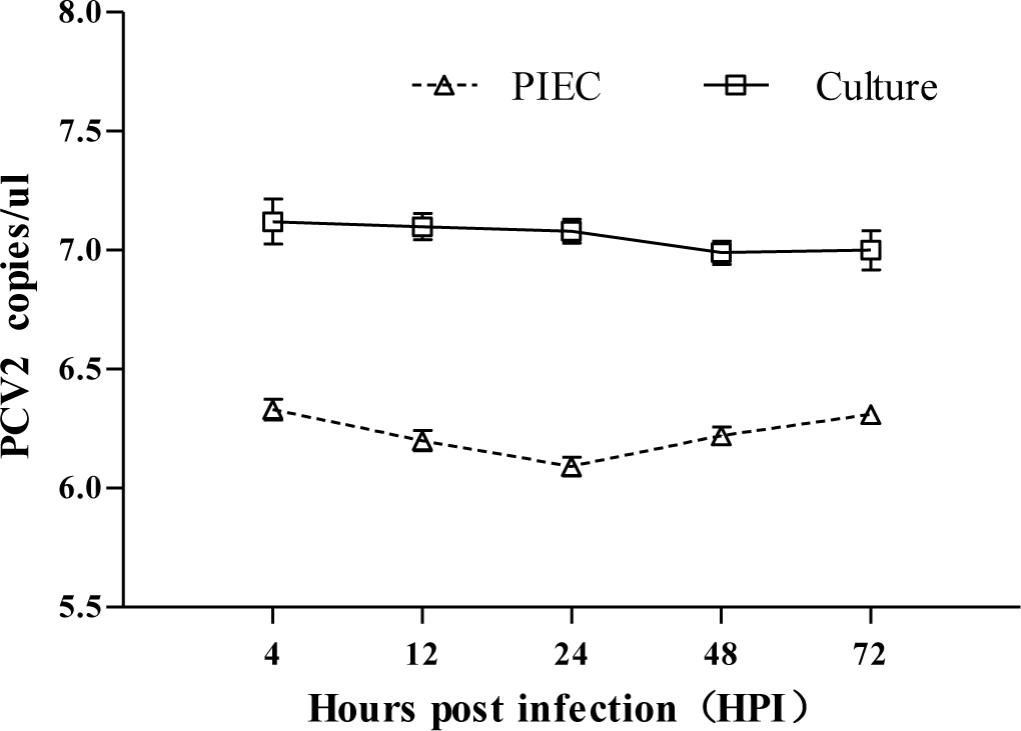
Viral loads of PCV2 in PIECs and culture. The abundance of PCV2 DNA was determined by FQPCR and expressed as the mean of the PCV2 DNA copy number. Δ: viral load in PIECs, □: viral load in the mixture of PIECs and supernatants. Error bars represent the standard deviation. The data are shown as the mean ± standard deviation values from three independent experiments.

As shown in Fig 1, the viral load in PIEC cultures decreased slightly from 4 to 72 h. Moreover, the viral load in PIECs decreased from 4 to 24 hpi and then increased slowly from 24 to 72 hpi.

### Effect of PCV2 infection on apoptosis and death of PIECs

**Fig 2:**
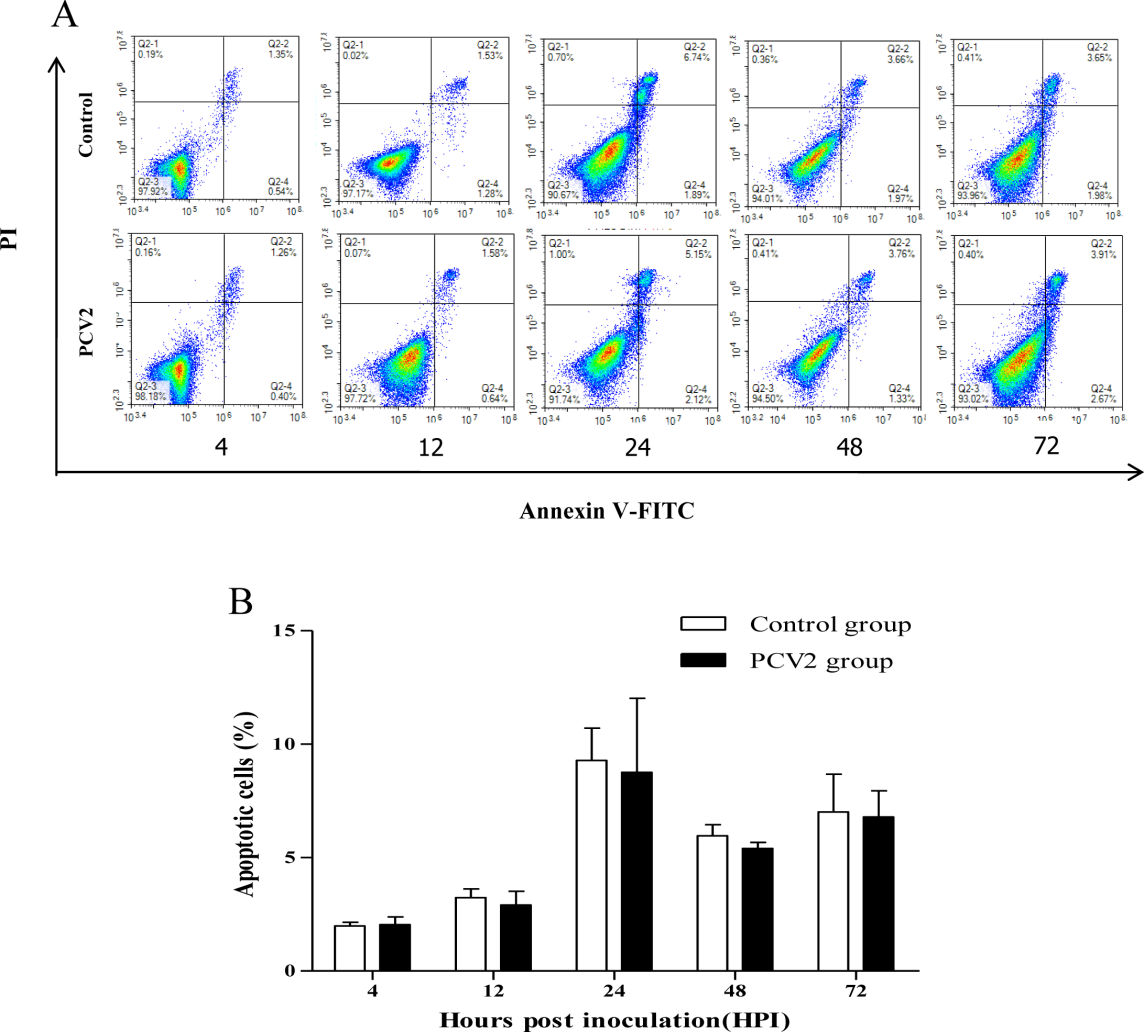
Effect of PCV2 infection on apoptosis of PIECs. Analysis of apoptosis of PCV2-infected PIECs and control (untreated) PIECs at different post-infection time points. (A) Annexin V-FITC/PI dot plots of PIECs at different hours post infection, as determined by flow cytometry. (B) Percentage of apoptotic PCV2-infected PIECs and untreated PIECs at different time points post infection. Data are presented as the mean percentage of apoptotic cells and standard deviation (error bars) values for each time point indicated (n = 3 per group).

As shown in Fig 2, compared to the control, the percentage of apoptosis in PCV2-infected PIECs was not significantly different at 4, 12, 24, 48, and 72 hpi.

### Changes in the levels of IL-8 in PCV2-infected PIECs

**Fig 3:**
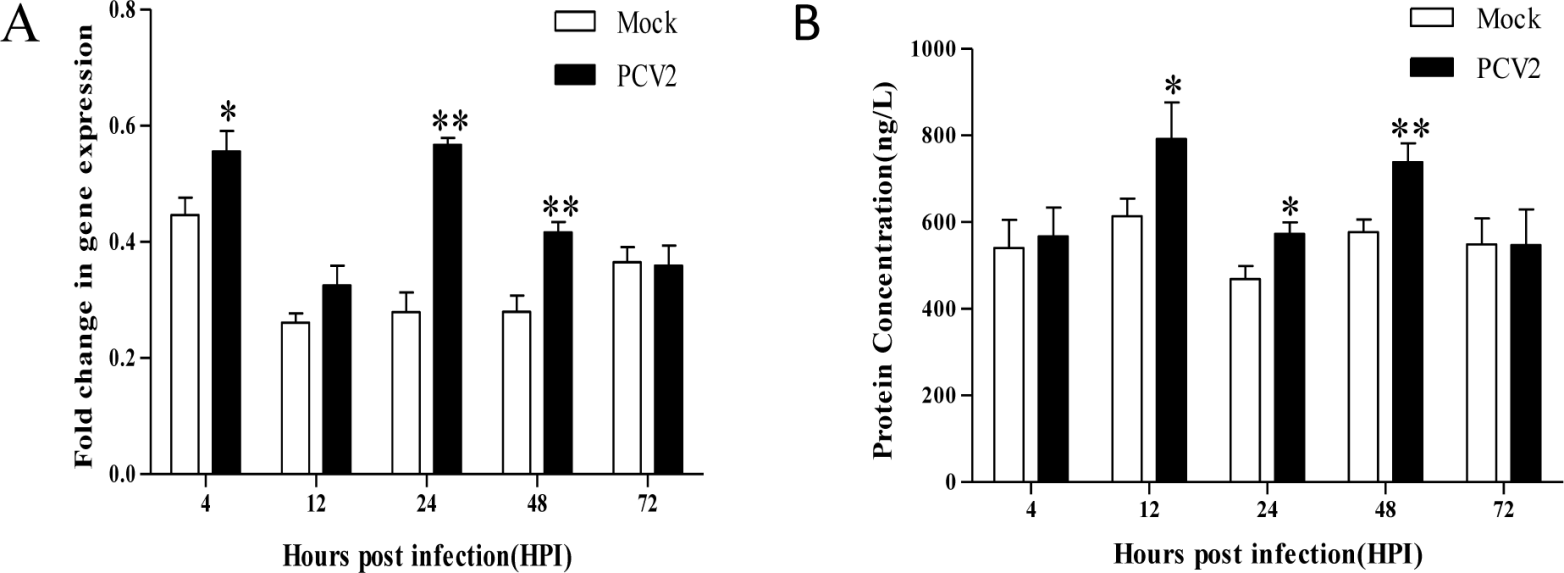
Changes in IL-8 expression in PIECs infected with PCV2. (A) Levels of IL-8 mRNA expression as detected by real-time QPCR. (B) Culture supernatant concentration of IL-8 as determined by ELISA. “Mock” and “PCV2” indicate the control PIECs and the PCV2-infected PIECs, respectively. Data are presented as the mean cytokine concentration and standard deviation (error bars) for each time point indicated (n = 3 per group). *P < 0.05 compared to the control, **P < 0.01 compared to the control.

The expression levels of IL-8 in PIECs infected with PCV2 were determined at the mRNA (real time FQPCR) and protein (ELISA) levels respectively. IL-8 mRNA expression levels were upregulated in PCV2-infected PIECs, with a significant difference (*P* < 0.05) at 4, 24, and 48 hpi compared to mock cells (Fig 3A). The protein expression level of IL-8 was significantly increased (*P* < 0.05) at 12, 24 and 48 hpi (Fig 3B).

### Effect of PCV2 infection on immune-related gene expression in PIECs at 24 hpi

**Fig 4:**
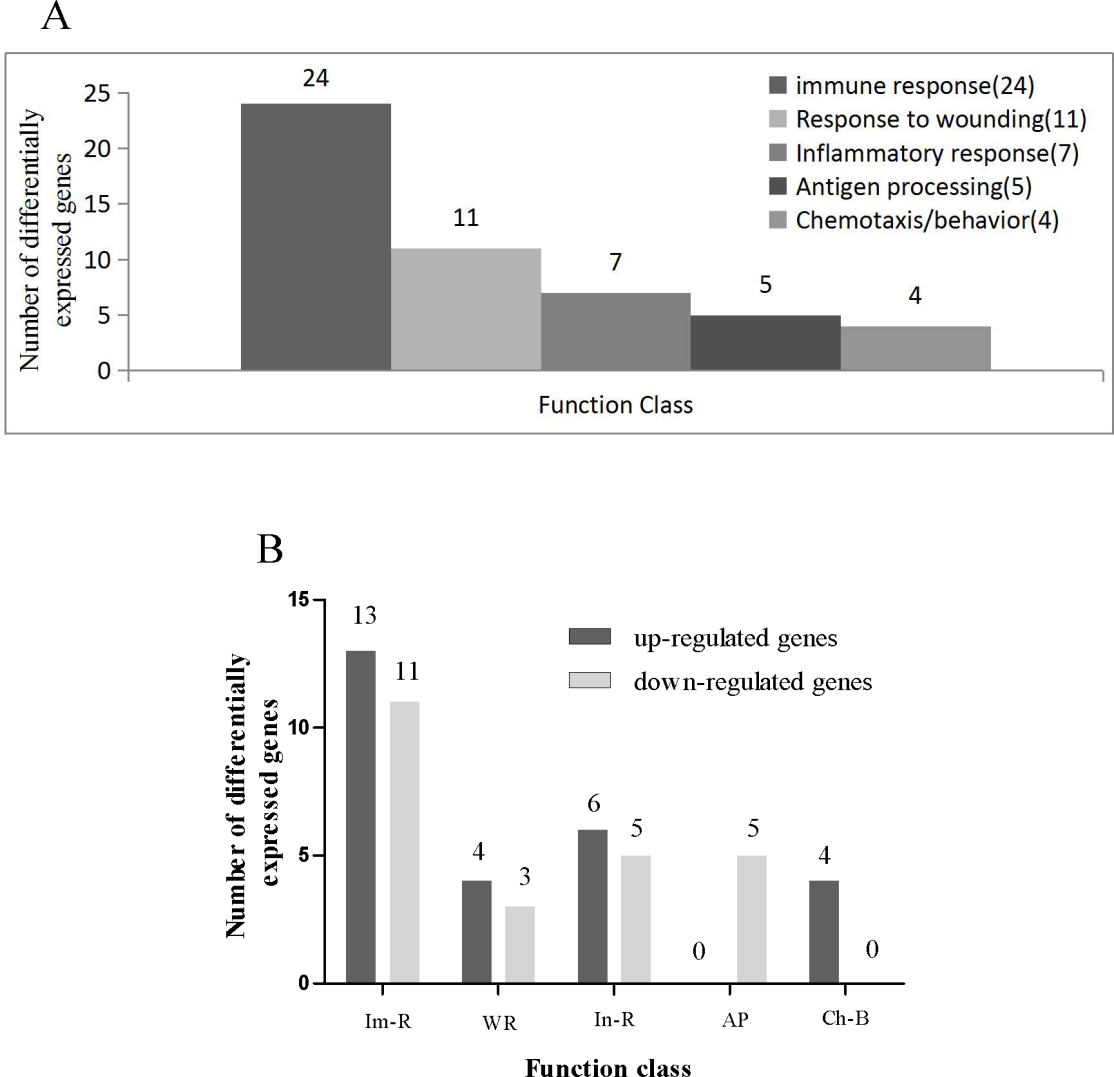
CHiP analysis of differentially expressed genes in PIECs infected with PCV2. (A) Genetic Ontology functional classification of differentially expressed genes in PIECs infected with PCV2. Functional annotations of 51 differentially expressed genes were obtained through GO analysis. A total of 51 genes that were differentially expressed between PCV2-infected and uninfected PIECs were selected for analysis. The numbers indicate the number of genes in each class that are differentially regulated. (B) Comparison of the function of genes that were upregulated or downregulated in PIECs infected with PCV2. The number of upregulated or downregulated genes is shown.

According to the dynamic changes in nucleic acid expression and the changes in the related cytokines in PIECs after PCV2 infection, PIECs at 24 hpi were selected for characterizing the changes in immune-related PIEC gene expression using a microarray approach. Genes with more than two-fold change (P < 0.05) in their expression in PCV2-infected and PCV2-uninfected PIECs are shown in Table 2. The analysis software DAVID, a comprehensive online resource that contains annotations of gene function, was used to identify the function of each differentially expressed gene. Gene ontology (GO) analysis indicated that 51 differentially expressed genes were involved in immune responses, inflammation, injury repair, molecular expression of antigen processing and presentation, and chemotaxis/behavior (Fig 4A and 4B; Table 2). With regard to the immune response genes, upregulation of 13 genes was observed to positively regulate innate immune response and antiviral activity, and downregulation of 11 genes was observed to negatively regulate immune response. With regard to the inflammatory response genes, increase in the expression of 4 genes and decrease in the expression of 2 genes was found to positively regulate inflammatory reactions. With regard to the response to wounding, increase in the expression of 6 genes and decrease in the expression of 4 genes was found to positively regulate inflammatory wounding. Further, downregulation of 5 genes was found to negatively regulate antigen processing and presentation ability, and upregulation of 4 genes was found to positively regulate chemotaxis/behavior.

**Fig 5:**
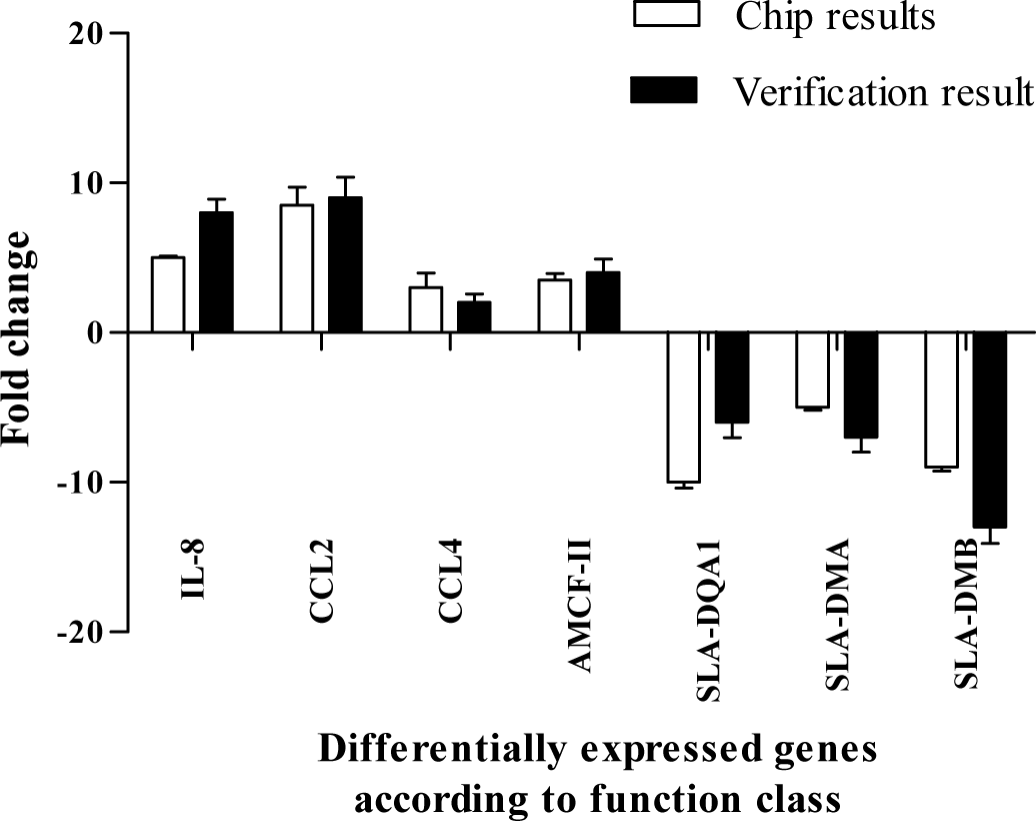
Verification of the results with real-time qPCR. A comparison of real-time quantitative PCR results (n = 3 per group) and gene chips results was performed. The fold change in gene expression in PCV2 infected PIECs compared to control PIECs is shown. Error bars represent the standard deviation.

A selected subset of genes that had at least two-fold changes in gene expression identified using the microarray were also assayed by real-time FQPCR. The results were consistent with the microarray analysis (Fig 5).

### Effect of PCV2 infection on PIEC migration

**Fig 6:**
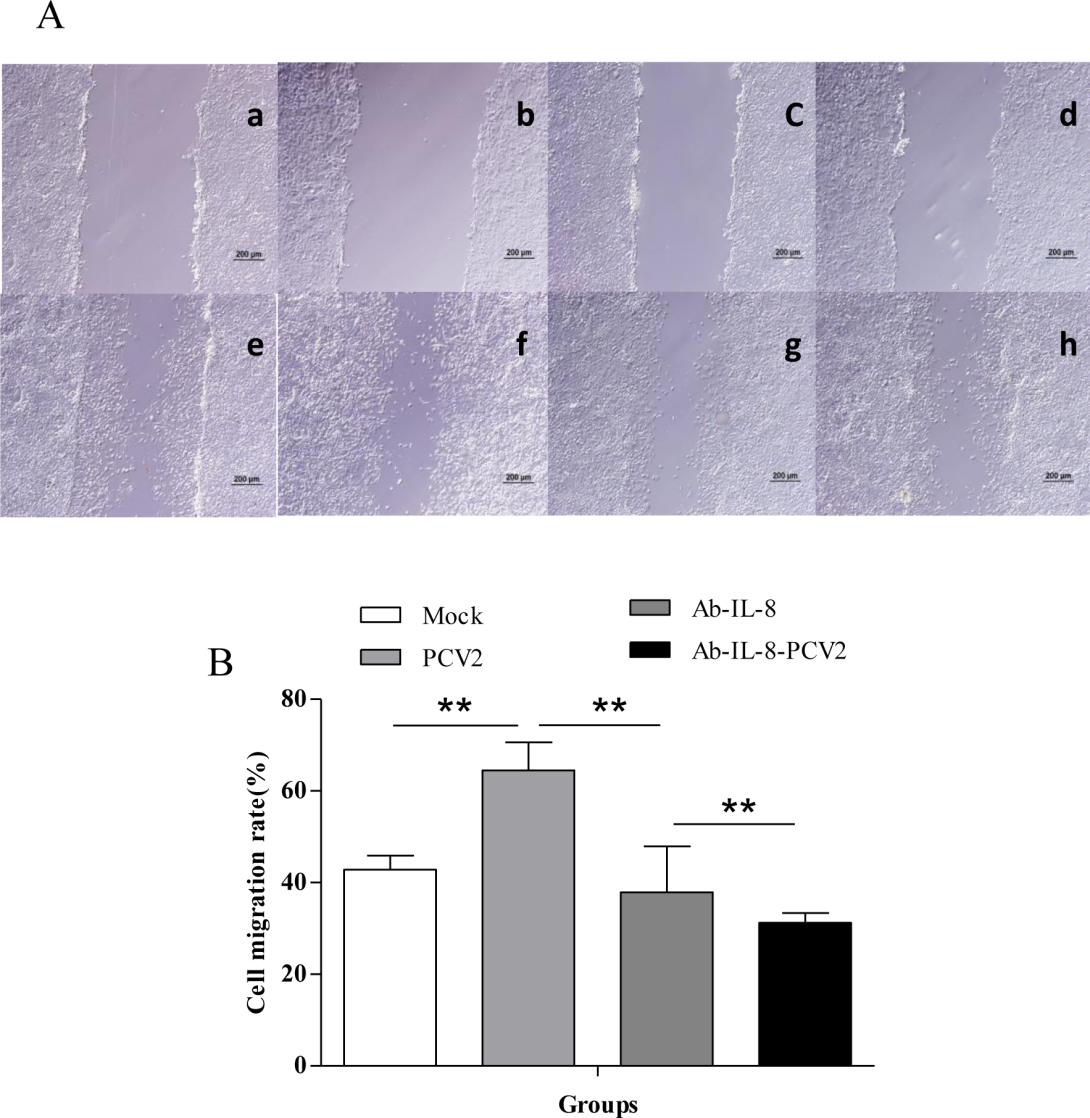
Changes in PIEC migration following PCV2 infection. (A) Representative images of the wound healing model. The width of the scratch is shown for the (a) control group, (b) PCV2-infected group, (c) IL-8 antibody group, and (d) PCV2-infected and IL-8 antibody group at post-wounding 0 h. PIEC migration into the scratch was determined 24 h later in the (e) control group, (f) PCV2-infected group, (g) IL-8 antibody group, and (h) PCV2-infected+IL-8 antibody group. (B) Changes in the endothelial cell migration rate following PCV2 infection. Error bars represent standard deviation. *P < 0.05, **P < 0.01. The data are shown as the mean ± standard deviation values of three independent experiments.

Since microarray results of PCV2-infected PIECs showed the upregulation of IL-8 expression and GO functional classification indicated that IL-8 has a wide range of functions, and QRT-PCR and ELISA also showed dynamic upregulation of IL-8 in PCV2-infected PIECs, we first assessed whether PCV2 or IL-8 impaired the ability of PIECs to repair a scratch wound in a cell monolayer by PCV2-infected PIECs and blocked IL-8 with a specific antibody. A representative example of the scratch wound and the PIECs migrating into the breach at 24 h after being wounded was shown for PCV2-uninfected PIECs (Fig 6A and 6E), PCV2-infected PIECs (Fig 6B and 6F), IL-8-antibody PIECs (Fig 6C and 6G) and PCV2-infected-IL-8-antibody PIECs (Fig 6D and 6H), respectively. On average, the PCV2-infected group had a significantly greater migration rate than the control group ((42.86 ± 3.05) % vs. (64.52 ± 6.09) %; *P* < 0.01). The addition of the IL-8 blocking antibody in the absence of PCV2 infection significantly reduced the migration rate compared to the untreated control group ((42.86 ± 3.05) % vs. (37.93±10.00) %; *P*< 0.01). In the context of PCV2 infection, addition of the IL-8 blocking antibody again significantly reduced PIEC migration into the scratch wound (PCV2 alone: (64.52±6.09) % vs. PCV2+antibody: (31.25±2.12)%; *P*< 0.01) (Fig 6I). These results indicated that PCV2 infection enhanced PIEC migration, while the IL-8 antibody inhibited its migration. Therefore, PCV2 infection, which upregulated the IL-8 expression of PIECs, likely promoted PIEC migration. However, other factors can also affect PIEC migration.

### Effect of PCV2-infected PIECs on DC maturation

Since some cytokines secreted by endothelial cells can inhibit DC maturation [31, 32], it is necessary to verify whether PCV2-infected endothelial cells further affect DC differentiation and maturation. Therefore, we used flow cytometry to assess changes in DC phenotype that might indicate functional changes.

**Fig 7:**
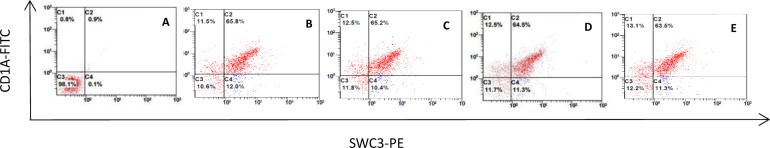
The CD1a/SWC3a dot plots of monocyte-derived DCs by flow cytometry analysis. Note: Representative flow cytometry plots showing CD1a and SWC3a staining in (A) monocyte-derived dendritic cells (DCs) alone, (B) DCs matured in co-culture with PIEC cells (PIEC-DC), and (C) DCs matured in co-culture with PCV2-infected PIECs (PCV2-PIEC-DC) are shown.

**Fig 8:**
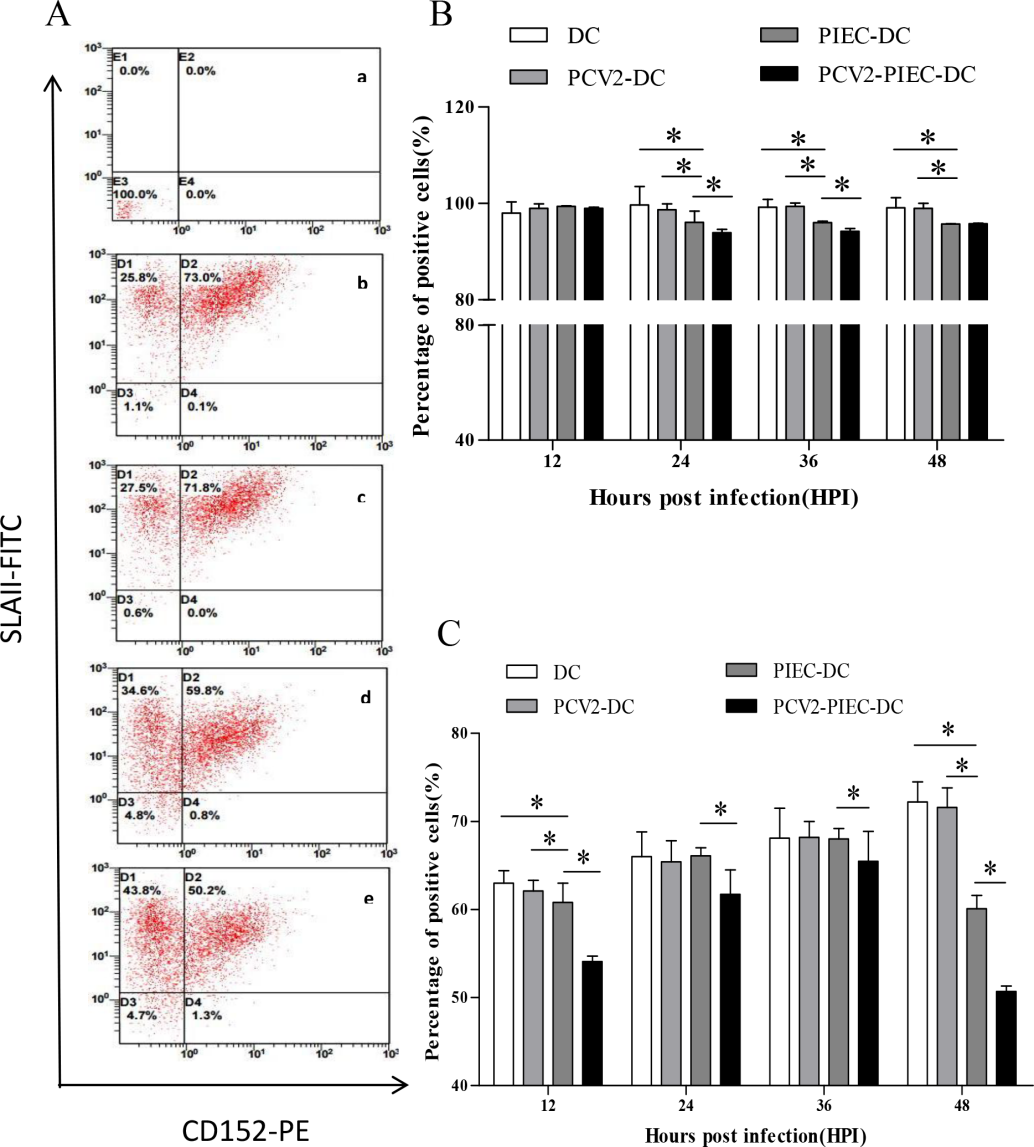
Dot plots and percentage of dendritic cells expressing the maturation markers MHC-II and CD80/86. (A) Flow cytometry analysis showing the expression of MHC II and CD80/86 in monocyte-derived DCs: (a) monocyte-derived dendritic cells (DCs) alone, (b) PCV2-infected DCs (PCV2-DCs), (c) DCs matured in co-culture with PIEC cells (PIEC-DCs), and (d) DCs matured in co-culture with PCV2-infected PIECs (PCV2-PIEC-DCs). Percentage of dendritic cells expressing the maturation markers MHC II (B) and CD80/86 (C) in monocyte-derived DCs.

DC was monitored based on CD1a and SWC3a expression. The percentage of CD1a^+^/SWC3a^+^ cells was not significantly different between the induced DC groups. The MHC-II and CD80/86 expression of DCs was used to monitor DC maturation. In the presence of GM-CSF and IL-4, more than 60% of the CD14^+^ monocytes became CD1a/SWC3a double-positive cells, indicating the induction of DC, regardless of PCV2 infection in the PIECs (Fig 7). However, when DC maturation was assessed, it was found that there were no differences between PCV2-DCs and DCs alone. The percentage of DCs expressing MHC-II was significantly lower in the groups co-cultured with PIECs than the DC alone and PCV2-DC groups at 24, 36 and 48 hpi (Fig 8B; *P* < 0.05). The percentage of DCs expressing MHC-II in the PIEC-DC group was significantly lower than that in the DC alone and PCV2-DC groups at 24 and 36 hpi (Fig 8B; *P* < 0.05). Moreover, compared to the PIEC-DC group, expression of MHC-II in DCs in the PCV2-PIEC-DC group was downregulated significantly at 24 and 36 hpi (Fig 8B, *P* < 0.05). Except at 24 hpi, expression of CD80/86 in the PIEC-DC group and PCV2-PIEC-DC group was lower than that in the DC alone and PCV2-DC groups, with a significant difference observed at 12 and 48 hpi (Fig 8C, *P* < 0.05). Compared to the PIEC-DC group, the percentage of cells expressing CD80/86 was significantly decreased in the PCV2-PIEC-DC group at 12, 24, 36, and 48 hpi (Fig 8C, *P* < 0.05). All these findings indicate that PCV2-infected PIECs further inhibited DC maturation.

### Effect of PCV2-infected PIECs on the endocytic activity of DCs

**Fig 9:**
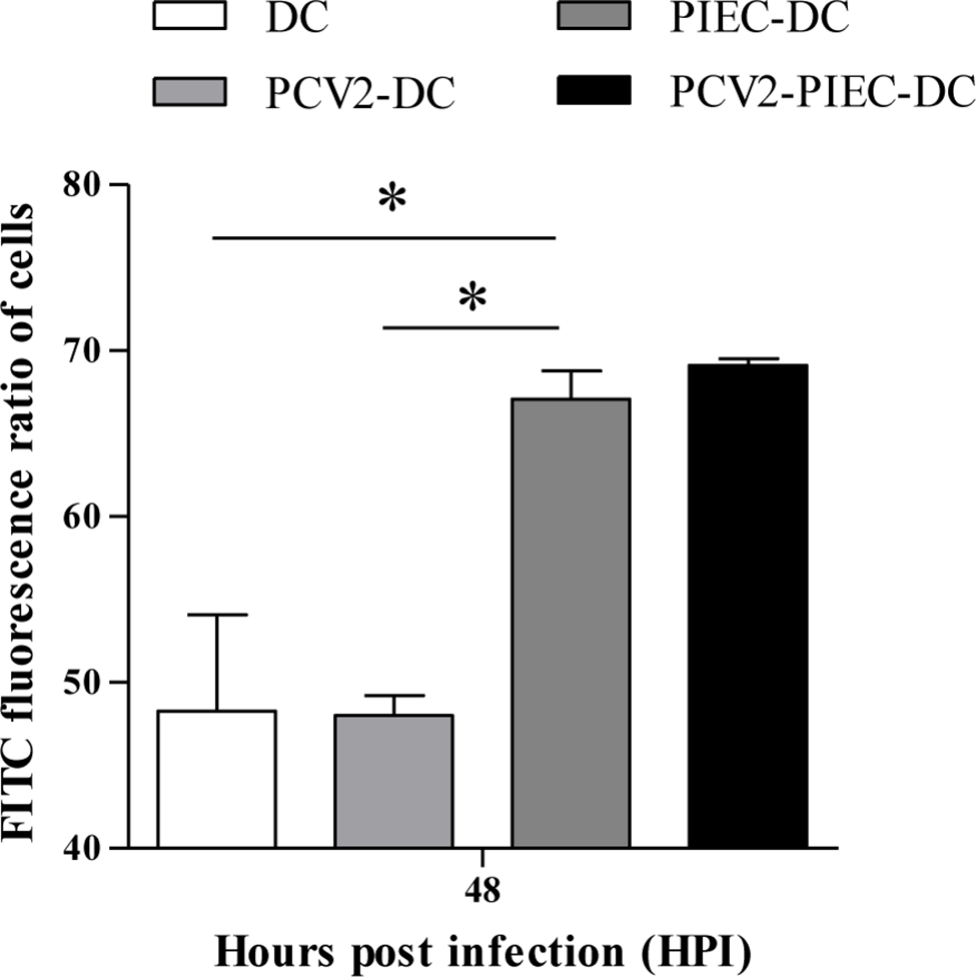
Endocytic analysis of DC. DCs that were induced using different culture methods were co-cultured with FITC-dextran 40,000 for 1 h at 37°C. The endocytosis percentage was evaluated by flow cytometry. Data are expressed as the average endocytosis percentage in each group (n = 3 for each group). Error bars represent the standard deviation. *P < 0.05 between two groups.

Given that PCV2-infected PIECs inhibited DC maturation, particularly at 48 hpi, in the PIEC-DC and PCV2-PIEC-DC groups compared to the DC alone and PCV2-DC groups, the endocytic activity of the DCs was measured at 48 hpi. The DCs from four groups were incubated with FITC-Dextran for 1 h respectively and then analyzed by flow cytometry. The percentage of FITC+ cells was significantly (P<0.05) greater in the PIEC-DC group (67.1±1.7) % and the PCV2-PIEC-DC group (69.11±0.4) % than in the DC alone group (48.3 ± 5.8) % and PCV2-DC group (48.0 ± 1.2) % (Fig 9). Furthermore, the percentage of FITC+ cells in the PCV2-PIEC-DC group was greater than that in the PIEC-DC group, but the difference was not significant. These results suggested that PIECs induced an increase in the endocytic activity of DCs, and that PCV2-infected PIECs enhanced the capacity of DCs to a greater extent.

### Effect of PCV2-infected PIECs on the stimulatory effect of DC on T lymphocytes

**Fig 10:**
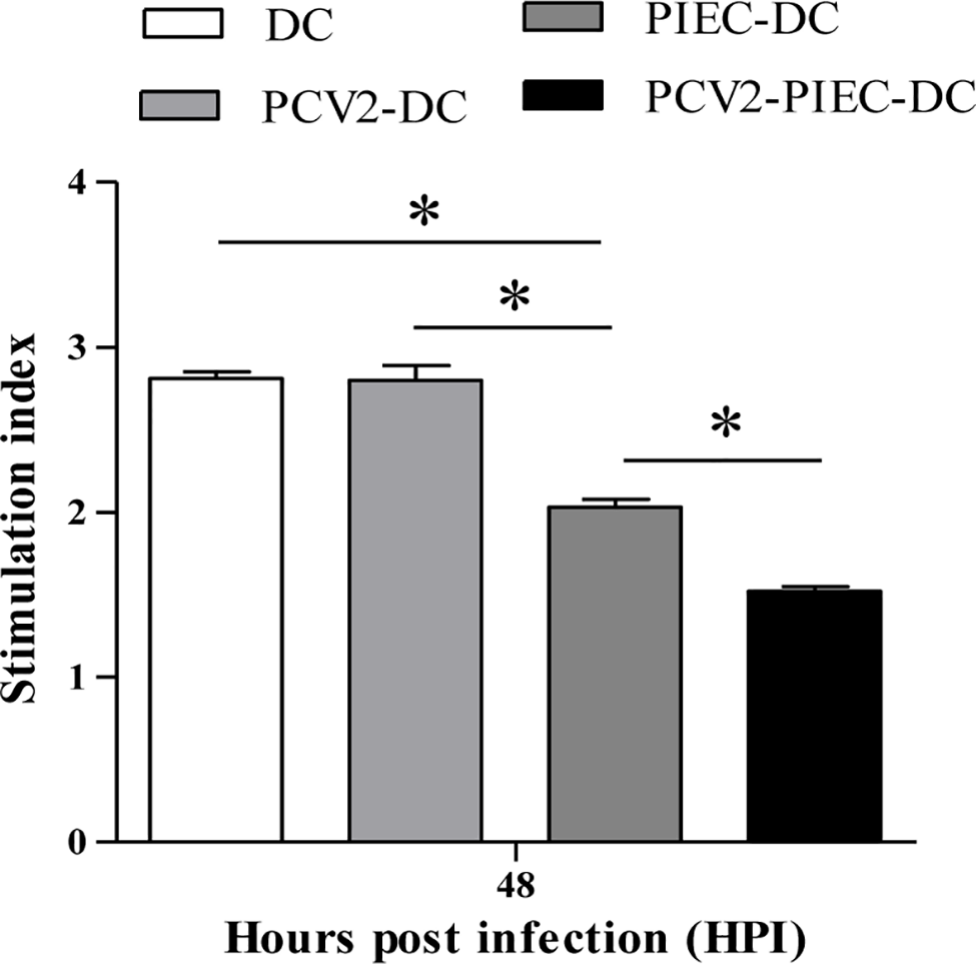
Stimulation index of autologous mixed lymphocyte reaction (AMLR)The SI in each group is shown. Data are expressed as the average SI in each group (n = 3 per group). Error bars represent the standard deviation. *P < 0.05.

Induced DCs from the PCV2-PIEC-DC, PIEC-DC, DC and PCV2-DC groups were co-cultured with isolated T cells in 96-well plates at a 1:10 ratio to determine whether the DCs had different T-cell stimulatory functions. The stimulation index (SI) was compared between groups. There was no difference between the DC group and PCV2-DC group (Fig 10). Compared to the DC group (2.81 ± 0.04) % and PCV2-DC group (2.8 ± 0.09) %, the SIs of the PIEC-DC (2.103 ± 0.05) % and PCV2-PIEC-DC (1.52 ± 0.03) % groups were significantly lower (P < 0.05) (Fig 10). Notably, the SI of the PCV2-PIEC-DC group was also lower than that of the PIEC-DC group. Based on these results, we concluded that PCV2-infected PIECs further inhibited the stimulatory effects of DCs on T lymphocytes and that PCV2-infected PIECs reduced the antigen-presenting abilities of DC.

## Discussion

This study was undertaken to understand the effects of PCV2 infection on PIECs and the possible secondary consequences for DC maturation and function. PCV2 infection reduced the immune response ability of PIECs, and promoted the inflammation and migration ability of PIECs. In addition, PCV2-infected PIECs further inhibited DC maturation, increased DC endocytic capacity, and reduced their ability to present antigens. These findings might suggest a pathogenic mechanism for PCV2.

Immune response and antigen presentation: the present study showed that PCV2-infected PIECs could enhance the immune response through stimulation of the complement system, upregulation of innate immune response, cellular immune response and NK cell cytotoxicity, degradation of viral mRNA, etc. Because formation of C1 is the first step in the classical complement activation pathway and enhances the immune response [14]. DDX58 (RIG-1), OAS, IL-1, IL-12 and IL-18 can augment the immune response and have antiviral effects [15–18, 33] by sensing viral infection, degrading viral mRNA during viral replication [33] or increasing NK cytotoxicity [17, 18]. In addition, IL-18 can also maintain and develop the inflammatory pannus by inducing endothelial cell migration and angiogenesis [19]. Moreover, VECs are non-specialized antigen-presenting cells that can express antigen-presenting molecules [34, 35]. Following PCV2 infection, decrease of antigen presenting molecules (SLA-DMA, SLA-DQB1, SLA-DQA1, SLA-DRB1, SLA-DMB), genes of antivirus (CD70 and GBP1) [36, 37] and innate immune (Toll-like receptor 1, TLR1) [38] indicated that PCV2 infection might reduce the antigen presenting and innate immune functions of PIECs. Downregulation of these genes might contribute to immune suppression, secondary infection and vaccination failure caused by PCV2 in swine [5, 6]. Moreover, type I IFN changes in PIEC-infected cells were not obvious compared to the control (data unshown), indicating that type I IFNs had little effect on DC maturation. All these findings may explain why the viral nucleic acid load of PIECs tends to be stable during the 72 h of viral infection, and there is no apoptosis of PIECs.

Inflammatory and wounding response: The chemokines (IL-8, AMCF, CCL2, CCL4) and the complement protein C5 can promote inflammation by different ways [39–43]. Moreover, endothelial IL-8 can also ensure firm adhesion of monocytes to the endothelium [44, 45] [39, 40] and exert the damage to the blood vessel [46]. ITIH4 can rapidly induce a nonspecific defense reaction to different stimulus [47]. Fetuin exerts anti-inflammatory effects [48]. THBD and CFB positively regulate inflammatory injury and lead to complement activation and alternative pathways of complement activation [49, 50]. In present study, the increase in complement system initiation factors (C1s), chemotactic factors and acute phase proteins, and decrease of anti-inflammatory factors positively regulated the damage response and inflammatory response caused by viral infection. Decrease of C5 and inflammatory acute phase proteins negatively regulated the inflammatory response and damage response. These two effects may result in the rapid repair of PCV2-infected PIECs after injury. At the same time, the accumulation of monocytes, macrophages and neutrophils caused by inflammatory factors and chemokines may lead to vasculitis and even granuloma formation [5].

Notably, PCV2 infection upregulated IL-8 expression in PIECs, the functions of which are reflected in the immune response, inflammatory response, chemotaxis, and damage response. To understand the effects of PCV2 infection and upregulation of IL-8 on PIEC migration and injury repair, a cell culture model was established for wound healing by scratching a cell monolayer. EC migration in vessel walls represents an essential phase of the wound healing process following injury and loss of this function can lead to severe loss of function for the endothelium [51]. The PIEC migration assay showed that PCV2 infection contributed to PIEC migration by upregulating the expression of endothelium-derived IL-8, suggesting a mechanism for granuloma formation in many different organs and tissues during porcine circovirus disease (PCVD) [5].

Our data showed that the major histocompatibility complex (MHCII) and the costimulatory molecule CD80/86 expression on induced DCs were reduced in all co-culture groups accompanied by the increased ability to engulf macromolecules and the decreased ability to stimulate T cells, but these abilities were further amplified after co-culture with PCV2-infected PIECs, suggesting that the changes in endothelial cell function caused by PCV2 infection can inhibit the maturation of DCs and induce immunosuppression. For immature DCs that uptake antigens in the peripheral blood pass through the lymphatics and reach the lymphatic vessels following interaction with endothelial cells, and finally present antigens to the target resting lymphocytes with the acquisition of the mature phenotype [52]. Mature DCs highly expressing MHC II and CD80/86 can promote differentiation of T lymphocytes [53]. Although PCV2-infected PIECs have upregulated secretion of IL-4, IL-10, IL-2 and IFN-ɣ [13], in the present study, PCV2 infection did not affect the expression of VEGF and IL-6 in PIECs, based on which it can be inferred that enhanced IL-8 expression of PIECs after PCV2 infection plays a key role in DC maturation. So, IL-8 changes in PCV2-infected PIECs are worthy of attention.
